# Intravital Microscopy Reveals Differences in the Kinetics of Endocytic Pathways between Cell Cultures and Live Animals

**DOI:** 10.3390/cells1041121

**Published:** 2012-11-16

**Authors:** Andrius Masedunskas, Natalie Porat-Shliom, Kamil Rechache, Myo-Pale’ Aye, Roberto Weigert

**Affiliations:** Intracellular Membrane Trafficking Unit, Oral and Pharyngeal Cancer Branch, National Institute of Dental and Craniofacial Research, National Institutes of Health, 30 Convent Dr. 303A, Bethesda 20892-4340, MD, USA; Email: andrius.masedunskas@gmail.com (A.M.); poratshliomn@nidcr.nih.gov (N.P.-S.); kamilrechache@rcsi.ie (K.R.); myopaleaye@gmail.com (M.-P.A.)

**Keywords:** intravital microscopy, *in vivo* imaging, endocytosis, cytoskeleton, salivary glands

## Abstract

Intravital microscopy has enabled imaging of the dynamics of subcellular structures in live animals, thus opening the door to investigating membrane trafficking under physiological conditions. Here, we sought to determine whether the architecture and the environment of a fully developed tissue influences the dynamics of endocytic processes. To this aim, we imaged endocytosis in the stromal cells of rat salivary glands both *in situ* and after they were isolated and cultured on a solid surface. We found that the internalization of transferrin and dextran, two molecules that traffic *via* distinct mechanisms, is substantially altered in cultured cells, supporting the idea that the three dimensional organization of the tissue and the cues generated by the surrounding environment strongly affect membrane trafficking events.

## 1. Introduction

The advent of GFP technology and fluorescent light microscopy has significantly changed cell biology [1,2]. Specifically, the ability to image subcellular structures has provided groundbreaking information in several fields such as membrane traffic, cell signaling and cell metabolism [1,2]. Recently, the introduction of super resolution microcopy has enabled imaging beyond the diffraction limit of light, thus opening a new era of investigations at the level of single molecules [3,4]. Most of these technologies have been applied to cells cultured on solid surfaces (e.g., glass or plastic). These versatile models allow proper control of the experimental conditions and can be easily manipulated both pharmacologically and genetically. However, one of their main limitations is the lack of the complex organization that is found in fully developed tissues [5,6]. Indeed, in tissues, cells are located in a three-dimensional environment and interact with both components of the extracellular matrix and other cells. On the other hand, cells grown on solid surfaces are engaged in interactions with the substrate only on one side, thus creating an artificial polarity. Finally, cells in a living tissue constantly receive cues from both the vasculature, the nervous systems and from other cells, which all contribute to their function. 

Imaging biological processes in live animals has been a daunting task. Although the surgical techniques to access the organs have been available for the past several decades, the lack of appropriate microscopes, light sources, optics, and probes have hampered the use of light microscopy for live animal imaging. In the early nineties, the first two-photon microscope was built by the Web laboratory enabling deep tissue imaging [7], and giving birth to a new field called intravital microscopy (IVM) [8,9,10]. Initially, IVM has been used in neuroscience to image synaptic plasticity in live animals, and later its use has been extended to study cell movement during immuno-response and cell migration during invasion and metastasis [3,4,6,8,9,11,12,13,14,15]. In the last few years, IVM has been used to image subcellular structures, such as nuclei [16], mitochondria [10], endosomes [17,18,19], and secretory granules [20,21]. The first time lapse images of endocytosis *in vivo* were performed in the kidney of live rats and mice where the internalization of fluorescently labeled dextrans and folate have been imaged in proximal tubuli [22,23]. However, these studies were limited to short time sequences due to the motion artifacts derived from heartbeat and respiration. More recently, our group has developed an experimental model based on the use of the salivary glands (SGs) as a model organ, which makes possible imaging at a subcellular resolution by minimizing the motion artifacts and extending the imaging time (see [24] for a detailed protocol). Using this experimental model we have investigated several aspect of membrane trafficking such as, regulated exocytosis of large secretory granules [20,25], endocytosis and trafficking of molecules through the endo-lysosomal system [17,26], and stimulated uptake of plasmid DNAs from the apical plasma membrane of the SG epithelium [18,27]. Interestingly, we found that both the regulation and the modality of exocytic events differed significantly between animal models and *in vitro* experimental systems [20,28]. Here, we sought to extend our investigations to endocytic events. Specifically, we imaged and compared the kinetics of uptake of transferrin (Tfn) and dextran in a population of stromal cells both in the SGs of live rats and after the cells were explanted and grown on glass. 

## 2. Results and Discussion

### 2.1. Cell Architecture and Organization of Endocytic Organelles Differ between Cell Cultures and Fully Developed Tissue

**Figure 1:**
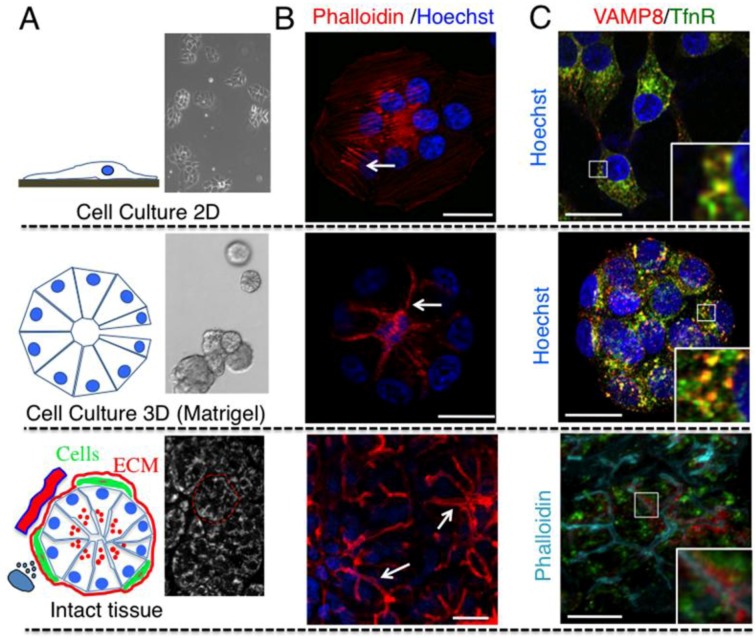
Cell architecture and organization of subcellular compartments depend on the experimental model—human submandibular glands (HSG) cells were cultured either on glass (2D, upper panels) or on 5 mg/mL matrigel (3D, center panels), as described in Material and Methods. (**A**) Phase contrast images of HSG cells grown in 2D (upper panel) or in 3D (center panel). In 3D cells form acinar-like structures that resemble the acini in intact SGs. In the lower panel an intact rat submandibular gland was imaged by two-photon microscopy (Excitation 740 nm) as previously described [17]. An acinus is highlighted (red broken line). (**B**) HSG cells grown in 2D or 3D and intact salivary glands were labeled with Hoechst (blue) and Texas-Red Phallodin (red) to highlight nuclei and actin respectively. Z-scans were acquired by confocal microsopy (excitation 405 nm and 562 nm) and maximal projections of the stacks are shown. In 2D, actin is mainly localized in stress fibers (upper panels, arrow), whereas in 3D is localized at the plasma membrane (center panel, arrow). In intact SGs, actin is localized primarily at the apical plasma membrane and decorates the acinar canaliculi (lower panel, arrows). Bars, 10 μm. (**C**) HSG cells and intact SGs were labeled with antibodies against VAMP8 (red) and TfnR (green). HSG cells were also labeled with Hoechst (blue), whereas SGs were labeled with phalloidin (Cyan). Note that in SGs VAMP8 is localized close to the apical plasma membrane and does not colocalize with TfnR (lower panel). Bars, 10 μm.

Cells cultured on solid surfaces are exposed to an environment that is different from the one surrounding cells in a living multicellular organism. In order to determine whether environment and spatial organization affect the architecture of subcellular compartments, we compared cells grown either on glass or in a 3D matrix with cells in intact tissues. Specifically, we used a cell line derived from human submandibular glands (HSG) (Figure 1A upper panel) [29,30] which forms polarized acinar-like structures that morphologically resemble the acini in intact SGs, when cultured in matrigel (Figure 1A center panel) [31,32]. First, HSG cells were stained with phalloidin, to reveal the organization of the actin cytoskeleton (Figure 1B). In HSG grown either on glass or plastic surfaces actin formed stress fibers and the nuclei where localized in the center of the cells (Figure 1B upper panel). When grown in matrigel, cells formed polarized acini with the nuclei located at the basolateral plasma membrane and actin reorganized in thick bundles at the apical cell cortex (Figure 1B middle panel, arrow). Notably, in intact SGs the organization of actin was more complex. Indeed, actin decorated very narrow canaliculi that are localized exclusively at the apical plasma membrane (Figure 1B lower panels, arrows). These results suggest that in SGs-derived cells removing the constrains imposed by the interaction with a solid substrate and establishing cell-cell interactions in three dimensions is sufficient to promote a dramatic rearrangement of the architecture of the cells, the actin cytoskeleton, and to initiate polarity. Similar conclusions were derived for mouse fibroblasts grown under similar conditions and compared with mouse tissue [33]. Next, we analyzed the localization of transferrin receptor (TfnR), a marker for early and recycling endosomes [34], and VAMP8, a SNARE protein that has been implicated in regulated exocytosis in several systems [35,36]. Interestingly, in both 2D and 3D cell cultures, VAMP8 colocalized with TfnR (Figure 1C upper and center panels), whereas in the intact tissue they did not. More specifically, in SGs, VAMP8 localized in the subapical area of the acinar cells, whereas TfnR localized in intracellular vesicles closer to the perinuclear area (Figure 1C lower panels). These findings strongly suggest that both the spatial organization and the unique combination of molecules that are present in the intact tissue substantially affect the organization of the intracellular compartments. 

### 2.2. The Dynamics of Endocytosis in Live Animals Differs from that in Cell Culture

**Figure 2:**
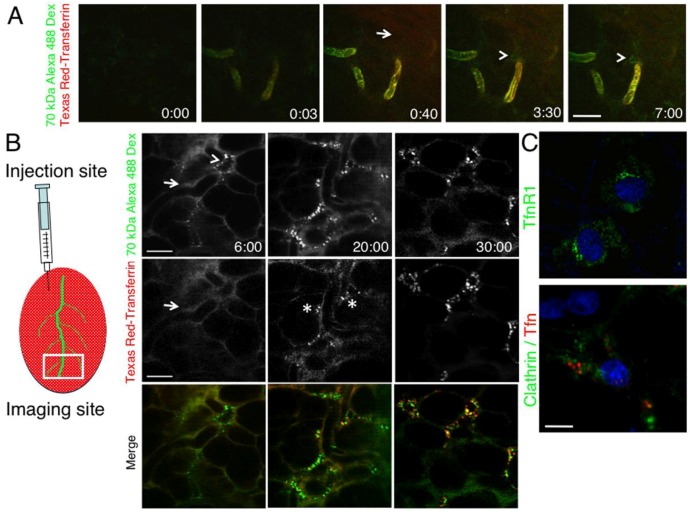
Kinetics of internalization of dextran and transferrin *in vivo*. (**A**) The submandibular SGs of an anesthetized rat were exposed and imaged in time-lapse by two-photon microscopy (excitation wavelength 840 nm). A mixture of 10 μg/mL TXR-Tfn and Alexa 488-dextran (70 kDa) was injected into the tail artery of the animal (time 0:00). After few seconds both probes appeared in the vasculature (time 0:03) and diffused into the stroma (time 0:40, arrow). After few min (time 3:30 and 7:00) only dextran (green) was internalized by stromal cells (arrowheads). (**B**) Left panel. Diagram showing the procedure for intra-organ injection. Right panel. A mixture of 10 μg/mL TXR-Tfn and Alexa 488-dextran (70 kDa) was injected into the stroma of the rat SGs and the internalization was imaged in time-lapse by intravital two-photon microscopy (Excitation wavelength, 840 nm). Snapshots were taken at 6, 20 and 30 min. Tfn in red and dextran in green. Bar, 20 μm. (**C**) Rat submandibular SGs were either fixed immediately (upper panel) and labeled for TfnR (green) and the nuclei (blue) or injected with TXR-Tfn (lower panel, red), and then processed for immunocytochemistry to reveal clathrin (green) and the nuclei (blue). Note, that at steady state TfnR1 is primarily localized in endosomal structures. The levels at the plasma membrane are low and not easily detectable by antibody staining. Bar 5 μm.

These morphological differences raise a fundamental question on whether the dynamics of membrane trafficking events may vary between cultured cells and live animals. Since we have recently shown that both the regulation and the modality of exocytosis in live animals are significantly different from what reported in *in vitro* model systems [20], here we sought to extend our investigation to endocytic events. We imaged fluorescently-labeled Tfn and dextran in the submandibular SGs of live rats, two molecules that are internalized by clathrin-dependent and clathrin-independent pathways, respectively [37,38]. To this aim, Texas-Red Tfn (TXR-Tfn) and Alexa-488 dextran (488-D) were injected systemically in the tail artery of anesthetized rats, as previously described [17,26]. Since excess of Tfn can be diverted to non-clathrin pathways, we injected 200 μg of fluorescent Tfn an amount equivalent to approximately 1:100 of the Tfn plasma levels, as estimated by others [39]. Moreover, in order to minimize any difference between the diffusion of the two probes either from the vasculature or throughout the stroma, we used a 70-kDa dextran that has a MW close to that of Tfn (80 kDa). We found that both molecules appeared in the vasculature and diffused into the stroma a few seconds after injection (Figure 2A, arrow). Three min after the injection only dextran was internalized in stromal cells (Figure 2A arrowheads) whereas Tfn was detected in endosomal vesicles after 10 min (not shown). Interestingly, endocytosis was evident mainly in stromal cells while the polarized epithelium did not seem to internalize the fluorescent Tfn and dextran. Furthermore, the observed Tfn internalization by stromal cells was slower than what has been reported in tissue culture. One explanation for the delayed internalization of Tfn with respect to dextran is a difference in the diffusion rate out of the vasculature, in spite of their similar MW. To overcome this potential issue we delivered the probes by a direct injection into the exposed glands, and imaged in an area distant from the injection site (Figure 2B). Interestingly, although both molecules were clearly observed in the stroma (Figure 2B left panels, arrows), only dextran was internalized by stromal cells within 5 min from the injection (Figure 2B left panels, arrowhead). After 20 min, Tfn was internalized and localized in endosomes different than those containing dextran (Figure 2B, center panels, asterisks). After 30 min, both molecules overlapped in a small subpopulation of endosomes (Figure 2B, right panels). Notably, the slower rate of Tfn internalization was not due to the lack of expression of TfnR and/or clathrin, as shown in Figure 2C, suggesting that *in vivo* Tfn uptake may be regulated differently than *in vitro*. 

**Figure 3:**
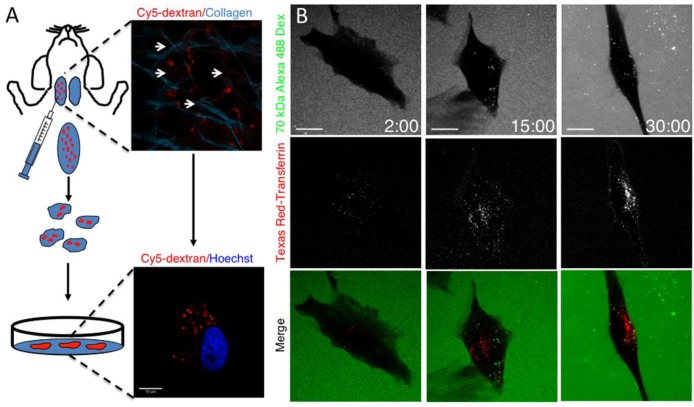
Kinetics of internalization of dextran and transferrin *in vitro*. (**A**) Diagram showing the procedure to label and isolate stromal cells from rat submandibular SGs. Anesthetized animals were injected with Cy5-dextran and euthanized after 1 h. The submandibular SGs were removed and imaged by two-photon microscopy (excitation 930 nm) to detect the internalization of the dextran (red) in stromal cells (upper panel, arrows). Collagen fibers were also imaged (cyan). The glands were minced and dispersed by enzymatic digestion. Cells were sorted by FACS and cultured on glass coverslips. To check whether cy5-dextran was retained in cultures, cells were labeled with Hoechst (blue) imaged by confocal microscopy (lower panel). (**B**) Rat derived stromal cellswere grown on coverslips for 24 h and incubated with a mixture of 10 μg/mL TXR-Tfn and Alexa-488 dextran (70 kDa). Cells were imaged in time-lapse by using confocal microscopy (excitation 488 nm and 561 nm). Snapshots were taken at 2, 15 and 30 min. Tfn (red) was internalized within the first 2 min (left panels) whereas dextran (green) appeared after 10–15 min (center and right panels). Bar, 5 μm**.**

Indeed, in cell culture Tfn has been reported to be internalized in few seconds, whereas dextran exhibits a slower kinetics of internalization [40]. To determine whether the kinetics of internalization observed in live animals is a unique property of the SGs stromal cells, we developed a procedure to isolate and culture them on glass. Specifically, a 70 kDa Cy5-dextran was injected in the SGs, and after one h they were excised, minced, and the stromal cells were enzymatically isolated, as described in the experimental procedures. Cells were then seeded on glass coverslips and the presence of dextran was assessed by confocal microscopy (Figure 3A). After 24 h, adherent cells were incubated with fluorescent 488-D and TXR-Tfn. Strikingly, Tfn was internalized within 30 seconds, whereas dextran was internalized after 10–15 min (Figure 3B). After 30 min, both probes were detected in intracellular structures and no colocalization was detected. 

These results suggest that endocytic pathways undergo substantial modifications when cells are removed from their *in vivo* environment. These modifications can be interpreted as an adaptive response to the new environment. For example, we can speculate that the increase in the kinetics of Tfn internalization may be related to a need to increase iron metabolism, as protection from either oxidative stress or bacterial infection. Indeed, the increase in oxygen levels and the lack of cytokines from the immune system may work as a trigger to alter these processes. Alternatively, it is possible that the interactions between cells and extracellular matrix *in vivo* may negatively regulate receptor-mediated endocytosis. Understanding the mechanisms underlying these modifications may provide novel information on the regulation of endocytosis under physiological conditions. 

## 3. Experimental Section

### 3.1. Fluorescent Probes

All the fluorescent molecules and the secondary antibodies were purchased from Invitrogen (Carlsbad, CA, USA). Mouse anti-clathrin and rabbit anti VAMP8 antibodies were from Abcam Inc. (Cambridge, MA, USA), mouse anti-TfnR antibody was from Zymed (Carlsbad, CA, USA). 

### 3.2. Animal Procedures

Sprague–Dawley male rats weighing 150–250 g were obtained from Harlan Laboratories Inc. (Frederick, MD, USA). The animals were acclimated for one week before used for the procedures. Water and food were provided *ad libitum*. All the experiments were approved by the National Institute of Dental and Craniofacial Research (NIDCR, National Institute of Health, Bethesda, MD, USA) Animal Care and Use Committee. The animals were anesthetized by an IM injection of a mixture of Ketamine and Xylazine (100 mg/kg an 20 mg/kg respectively) with additional injections as needed. Intravital microscopy and injection of the probes were preformed as described in [17,24]

### 3.3. Intravital and Conventional Microscopy

An IX81 inverted confocal microscope (Olympus, Melville, NY, USA) was modified to perform two-photon microscopy as described previously [26]. The excised glands and cultured cells were imaged in the inverted setting while time lapse imaging on the live animal was performed in the upright configuration using an objective inverter (LSM Technology Inc., Shrewsbury, PA, USA) [41]. For the time lapse imaging the acquisition speed was set to 0.3 frames/sec. All the images and movies were acquired using a UPLSAPO 60X NA 1.2 water immersion objective (Olympus). 

### 3.4. Image Processing

When needed, the background noise was reduced by applying to each image one or two rounds of a 2 × 2 pixel low-pass filter by using METAMORPH (Molecular Devices). Brightness, contrast and gamma correction were applied. Volume rendering was performed using IMARIS 5.6 and 6.0 64 bit (Bitplane). The final preparation of the images was conducted with ADOBE PHOTOSHOP CS. 

### 3.5. Cell Cultures in 2D and 3D

HSG cells [30] were cultured in Dulbecco’s modified Eagle’s medium/Ham’s F-12 (1:1) (Invitrogen, CA) in the presence of 5% fetal bovine serum, 100 U/mL penicillin, and 100 mg/mL streptomycin (Sigma-Aldrich, MO) at 37 °C in a 5% CO_2_ humidified atmosphere. 

Growth factor-reduced matrigel (Trevigen, MD, USA) was diluted in complete medium at the appropriate concentration (2.0–6.0 mg/mL), added to a 12-well tissue culture dish, and incubated for 1 h at 37 °C before culturing of the cells. 50 cells/mm^2^ were added onto the pre-solidified matrigel and grown for different times. Acinar cells were recovered from the matrigel by incubating in cell recovery solution (Trevigen, MD) for 30 min on ice. Acinar cells released from the matrigel were spread on glass coverslips and dehydrated at 37 °C for 15 min. Cells were then fixed in 2% formaldehyde/PBS, incubated first in blocking solution (10% FBS, 0.02% sodium azide in PBS) for 15 min, and later with various primary antibodies for 1 h at RT. Cells were then washed and incubated with the appropriate secondary antibodies for 1 h at RT. After three washes with blocking solution, nuclei were labeled by incubating the cells with Hoechst 33342 before mounting.

### 3.6. Whole Mount Immunocytochemistry

Isolated glands were fixed for 20 min in 0.05% Glutharaldehyde/4% formaldehyde in 0.2M Hepes pH 7.3 and then transferred for 1 h in 4% formaldehyde. The glands were sliced from the surface and fixed again in 4% formaldehyde for 30 min. The fixative was washed in PBS and the slices were blocked overnight at 4 °C in PBS, 10% fetal bovine serum, 0.04% saponin (blocking solution). The slices were incubated for 1 h with the primary antibody in blocking solution, washed 3 × 10’ in PBS and then incubated with the appropriate secondary antibody for 1 h at R.T. The samples were washed 3 × 10’ in PBS and then imaged by either two-photon or confocal microscopy.

### 3.7. Cell Cultures from Rat Salivary Glands

Sprague-Dawley rats were anesthetized, the submandibular SGs exposed, and 50 μL of 10 μg/mL Cy5 dextran were injected on the side of each gland. After one h, the glands were excised, placed in ice-cold 1% BSA in DMEM. The glands were then injected with the same medium using a syringe equipped with a 30 GG needle. After 5-10 injections the connective tissue was separated from the SGs lobules, collected, and incubated for 1 h at 37 °C in 2 mg/mL collagenase and 0.125 mg/mL dispase (Invitrogen). The tissue was homogenized by continuous pipetting with a 10 mL pipette and filtered through a sterile gauze to remove large fragments. The cells suspension was centrifuged at 2,000 rpm for 5 min and the pellet suspended in fresh DMEM containing 5% fetal bovine serum, 100 U/mL penicillin, and 100 mg/mL streptomycin (Sigma-Aldrich, MO, USA). The cells suspension was seeded on sterilized glass coverslips and grown at 37 °C in a 5% CO_2_ humidified atmosphere. After 24 h the coverslips were incubated for 30 min with 0.5% BSA in DMEM and the internalization of Tfn and dextran was performed in the same medium. 

## 4. Conclusions

So far, the gold standard for the study of cell biological processes has been cell cultures. For example, most of our knowledge about membrane trafficking events is derived from *in vitro* studies and the use of very sophisticated experimental approaches and techniques. Cell cultures models are an extremely powerful tool to tease out complex molecular machineries and they will continue to be the main workhorse for studying several aspects of cell biology. However, the recent introduction of IVM as a tool to image subcellular structures offers the opportunity to address basic cell biology questions in the live animal. Furthermore, it would allow us to challenge various paradigms, arising from tissue culture models, in the context of a fully developed organ. 
